# Complications in the use of peripherally inserted central catheter associated with peripheral intravenous therapy: retrospective cohort[Author-notes fn01b]

**DOI:** 10.1590/1518-8345.7173.4341

**Published:** 2024-09-23

**Authors:** Elizângela Santana dos Santos, Elaine Barros Ferreira, Fernanda Titareli Merizio Martins Braga, Amanda Salles Margatho, Paulo Sousa, Renata Cristina de Campos Pereira Silveira

**Affiliations:** 1 Universidade de São Paulo, Escola de Enfermagem de Ribeirão Preto, Centro Colaborador de la OPS/OMS para el Desarrollo de la Investigación en Enfermería, Ribeirão Preto, SP, Brazil; ^2^ Universidade de Brasília, Faculdade de Ciências da Saúde, Brasília, DF, Brazil; ^3^ Universidade Nova de Lisboa, Escola Nacional de Saúde Pública, Lisboa, Estremadura, Portugal

**Keywords:** Central Venous Catheterization, Intravenous Infusions, Nursing, Peripheral Catheterization, Patient Safety, Nursing Care

## Abstract

**Objective:**

to analyze the occurrence of difficulty in the peripheral insertion of the central catheter and the presence of complications in the use of this device in hospitalized adults who received peripheral intravenous therapy through a short peripheral intravenous catheter and to identify whether there is an association between peripheral intravenous therapy and the presence of complications in the use of the peripherally inserted central catheter.

**Method:**

retrospective cohort, with patients aged 18 years or over, in a tertiary teaching hospital, with a peripherally inserted central catheter, who had at least one previous short peripheral intravenous catheter. Data were analyzed using descriptive statistics and Poisson regression.

**Results:**

the sample consisted of 76 patients. There was an association between difficulty in the insertion procedure and number of punctures (p<0.01) and insertion in the external jugular vein compared to the upper limbs (p<0.01). The insertion site was also associated with the removal of the peripherally inserted central catheter due to complications in the robust analysis of variance (p=0.02). No associations were identified between: difficulty inserting the device and time on peripheral intravenous therapy (crude model p=0.23; adjusted model p=0.21); difficulty in insertion with administration of irritating and vesicant medication (crude model p=0.69; adjusted model p=0.53); complication in the use of peripherally inserted central catheter and time of peripheral intravenous therapy (crude and adjusted models p=0.08); and secondary migration of the catheter tip with the device insertion site (p=0.24).

**Conclusion:**

it was possible to identify secondary migration as one of the main complications, resulting in premature removal of the device. Furthermore, the greater the number of puncture attempts to insert the PICC, the greater the difficulty in inserting it. Insertion into the external jugular vein was recurrent, with a higher risk of removal due to complications in relation to the upper limbs.

## Highlights

**(1)** There was no association between PIT time and difficulty inserting the PICC.

**(2)** There was no association between PIT time and complications in PICC use.

**(3)** Secondary migration of the PICC tip was the most frequently reported complication.

**(4)** The external jugular vein was associated with difficulty in PICC insertion.

**(5)** The external jugular vein was associated with the presence of PICC complications.

## Introduction

 Infusion therapy consists of the infusion of fluids via vascular, intraosseous, subcutaneous or intrathecal routes. Specifically for patients who receive nutritional therapy, blood components or medications intravenously or vascularly, it is called intravenous therapy ^(^ 1 ^)^ . 

 To establish Peripheral Intravenous Therapy (PIT), Peripheral Intravenous Catheters (PIVC) are used. Of these, the Short Peripheral Intravenous Catheter (SPIVC) is the most used in hospitalized patients ^(^ 1 ^)^ . 

 It is estimated that 80% of hospitalized patients use SPIVC ^(^ 2 ^)^ . However, despite the benefits and indications, there are complications associated with the use of this device. These complications can be classified as local or systemic, such as phlebitis ^(^ 3 ^)^ , primary and secondary migration of the tip ^(^ 4 ^)^ , Deep Vein Thrombosis (DVT ^(^ 4 ^)^ , catheter damage, occlusion ^(^ 3 ^)^ and Catheter-Related Bloodstream Infection (CRBSI) ^(^ 1 ^,^ 5 ^)^ . 

 For patients who require administration of incompatible medications peripherally, intravenous therapy for a prolonged period of time or without conditions for peripheral puncture, the use of a Central Venous Access Device (CVAD) may be indicated, one of which is the Peripherally Inserted Central Catheter (PICC) ^(^ 1 ^)^ . 

 The PICC is a long, flexible catheter, which has specific indications for use, such as antibiotic therapy for more than six days, use of irritating or vesicant medications, extremes of pH and high osmolarity. It is inserted through a peripheral vein and achieves central positioning in the distal third of the cavoatrial junction ^(^ 1 ^)^ . 

 In Brazil, the PICC is mostly inserted by duly qualified nurses, and this practice is legalized by Resolution nº 258 of the *Conselho Federal de Enfermagem* (COFEN) ^(^ 6 ^)^ . However, the use and acquisition of this device in hospitals of the *Sistema Único de Saúde* (SUS) are still limited, due to the high cost and cultural aspects that include unfamiliarity with the catheter and recommendations by some professionals ^(^ 7 ^)^ . 

 The use of the PICC is described in the literature mainly for pediatric and neonatal populations ^(^ 8 ^-^ 11 ^)^ . Despite being considered a first-choice catheter, it often faces delays in its insertion, failing to be inserted at the correct time, which causes significant damage and depletion of the peripheral venous network in hospitalized patients ^(^ 12 ^)^ . 

 Given this challenging scenario, it is essential to understand the difficulties related to PICC insertion and the possible complications associated with the use of this device. This is important knowledge, especially because the nurse, when needing to insert it to guarantee safe and effective intravenous therapy, may be faced with limiting conditions of the peripheral veins as a factor that prevents adequate insertion ^(^ 1 ^,^ 13 ^)^ . 

Furthermore, understanding the factors that contribute to unsuccessful insertion or the incidence of complications related to PICC use can help the healthcare team act to reduce these situations. This, in turn, improves the quality of care provided and favors the safety of adult patients using PICC, as research with this population is scarce. Therefore, the objective of this study was to analyze the occurrence of difficulty in the peripheral insertion of the central catheter and the presence of complications in the use of this device in hospitalized adult patients who received previous peripheral intravenous therapy through a short peripheral intravenous catheter, as well as to identify whether there is an association between peripheral intravenous therapy and the presence of complications when using a peripherally inserted central catheter.

## Method

### Study design

Retrospective and longitudinal cohort study. The recommendations of the Strengthening the Reporting of Observational Studies in Epidemiology (STROBE) were used to report the research.

### Location

The study was carried out in a tertiary public teaching hospital in the city of Londrina, PR, Brazil, which has a committee of nurses trained and specialized in PICC insertion. This committee follows institutional protocols, but the choice of vein for insertion is carried out by the nurse who performs the procedure. There was no training for the committee’s nurses during the study period.

### Period

 Data were collected from January 1 ^st^ to December 31 ^st^ , 2020. 

### Population

Adults hospitalized during the data collection period who underwent a PICC insertion procedure, who had received prior peripheral intravenous therapy via a short peripheral intravenous catheter.

### Selection criteria

 All patients aged 18 or over, hospitalized between January 1 ^st^ and December 31 ^st^ , 2020, who had a PICC inserted successfully or unsuccessfully and preceded by at least one SPIVC were included. Those with previous insertion of a CVAD were excluded, as well as those who were discharged from hospital with the PICC for home use. 

### Sample definition

The sample was defined by convenience.

### Study variables

#### Dependent variable

*Occurrence of complications during PICC use:* occurrence of at least one adverse event during PICC use that did not result in device removal (primary or secondary migration, phlebitis, suspected infection, total occlusion, catheter damage, CRBSI, venous thrombosis, bleeding or skin injury) ^(^ 1 ^)^ . 

#### Independent variables

*PIT time (main variable):* duration, in days, from the first day of IPCIV use by the hospitalized patient until the date of PICC insertion; 

*Primary migration:* situation in which the PICC tip is not positioned in the superior vena cava or cavoatrial junction ^(^ 1 ^)^ ; 

*Secondary migration:* also called displacement, is the total exit of the PICC tip from the vein ^(^ 1 ^)^ ; 

*Phlebitis:* occurrence of one or more symptoms (pain and/or sensitivity at the insertion site and in the PICC route, erythema, edema, induration, purulent drainage or palpable venous cord) ^(^ 1 ^)^ ; 

*Suspected infection:* local or systemic symptoms of infection attributed to PICC without confirmation of CRBSI ^(^ 1 ^)^ ; 

*Total occlusion:* one or more lumens of the catheter present intraluminal obstruction, making it impossible to inject liquids or aspirate blood ^(^ 1 ^)^ ; 

*Catheter damage:* signs of visibly fractured catheter, local leak, catheter dysfunction, radiographic evidence of extravasation or tissue infiltration ^(^ 1 ^)^ ; 

*CRBSI:* primary bloodstream infection in a patient who had a central venous device within 48 hours before the development of the bloodstream infection without being related to an infection elsewhere ^(^ 1 ^)^ ; 

*Venous thrombosis:* in this study, DVT was considered, defined by the occurrence of thrombi visualized through imaging tests at the location of the PICC route in a patient with symptoms of pain in the catheterized limb, edema or redness ^(^ 1 ^)^ ; 

*Bleeding on insertion:* presence of visible blood at the device insertion site ^(^ 1 ^)^ ; 

*Skin injury:* presence of one or more symptoms (redness for more than 30 minutes after removing the dressing, skin laceration, skin peeling, skin blisters, trauma lesions, pustule, vesicle, papule or skin break) ^(^ 1 ^)^ ; 

*PICC use time:* PICC use time is considered, duration in days from device insertion to removal; 

*Difficulty inserting the PICC:* description by the nurse who performed the insertion of the PICC in the medical record or in a specific form about the presence of any difficulties in the insertion procedure; 

*Number of punctures:* number of punctures in the PICC insertion procedure; 

*PICC removal:* last day of PICC use, date of removal; 

*Inpatient unit:* place of hospital admission at the time of PICC insertion (emergency room, adult Intensive Care Unit (ICU), medical surgical unit, surgical center, burn treatment center and burn ICU); 

*Peripheral venous punctures prior to PICC* : number of SPIVC during PIT; 

*Vesicant and irritant medications used in PIT* : vesicant medication is considered to be one capable of causing blisters, peeling or necrosis when there is extravasation ^(^ 1 ^)^ ; 

*PICC insertion site:* PICC insertion site, the basilic, cephalic, axillary or other veins were classified as upper limbs, and external jugular; 

*PICC removal caused by complication* : primary or secondary migration, phlebitis, suspected infection, catheter damage, total occlusion, CRBSI, DVT, bleeding or skin injury that culminated in the removal of the PICC. 

### Instruments used to collect information

A form prepared by the author’s research group was used, with information about PIT, conditions of the venous network, multiple peripheral punctures, use of vesicant medications and patient characterization to collect data from the medical records. In addition to this, a PICC insertion procedure form was used, with information on the indication for insertion of the device, PICC data (material, caliber, lumens and length), insertion site, accessed vessel, venous network conditions that make it difficult to puncture the vessel and insert the catheter, difficulty in insertion, control radiography and record of ultrasound use and data on removal (reason for removal, integrity of the catheter, catheter tip cultures and blood culture).

### Data collection

 In data collection, patient records and PICC insertion procedure forms were consulted for those hospitalized from January 1 ^st^ to December 31 ^st^ , 2020. The PICC insertion procedure forms were filled out by nurses from the hospital infusion therapy committee. 

### Data processing and analysis

Initially, the data were described using absolute frequencies and percentages (qualitative variables) and through measures such as mean, standard deviation, minimum, median and maximum (quantitative variables). To estimate the Relative Risk for the outcomes of difficulty in insertion, complications during hospitalization, displacement and removal of the PICC in relation to the variables of interest, the Poisson regression model with simple and multiple robust variance (when appropriate) was used. The Log-rank test was applied to verify evidence of differences between survival curves. The analyzes were carried out using the SAS 9.4 software. In this work, a significance level of 5% (p < 0.05) was adopted and the Kolmogorov-Smirnov or Shapiro-Wilk tests were applied for normality and homoscedasticity.

### Ethical aspects

 The study was submitted and approved by the Research Ethics Committee (CEP, for its acronym in Portuguese) of the *Escola de Enfermagem de Ribeirão Preto* (EERP) and by the CEP of the *Hospital Universitário de Londrina* (HUL), under opinion nº 3.627.165 and CAAE 16451019.8.0000.5393, as per Resolution nº 466/2012 ^(^ 14 ^)^ . Waiver of the Free and Informed Consent Form was requested and obtained. 

## Results

### Participants

Of the 105 hospitalized adult patients who underwent PICC insertion, 28 were excluded because they did not have at least one SPIVC before PICC and another was discharged for home treatment with the device. The final sample comprised 76 patients, of which 59 (77.6%) had PICC insertion performed successfully and 17 (22.3%) unsuccessfully.

The average age at admission was 50 years (standard deviation 18.29), a minimum of 18 and a maximum of 90 years (median of 47 years), while the most prevalent gender was male (69.74%).

Regarding hospitalization units, more than half (51.3%) were in medical surgical unit. A particularity of this study is hospitalization in the Burn ICU, which reached 15.8%. Then, 21.0% of patients were admitted to the adult ICU.

#### Peripheral intravenous therapy before PICC

The average time in days that patients used SPIVC was 10.53 (standard deviation 31.58), with a median of 4 days, and a maximum of 274 days. The average number of venous punctures prior to PICC was 4.22 (standard deviation 3.74), median of three, at least one and maximum of 8.

The use of irritating and vesicant medications during this period was also analyzed, revealing that 72.3% of participants used this type of medication. The most recurrent were: Potassium Chloride (48.2%), Vancomycin Hydrochloride (29.0%) and Piperacillin sodium + Tazobactam sodium (25.4%).

There was no association between PIT time (with each increase of one day) compared to the difficulty in PICC insertion in the crude model (RR 0.99; 95% CI 0.98-1.00; p-value = 0.23) and adjusted model for gender and age (RR 1.00; 95% CI 0.99-1.00; p-value = 0.21).

For the administration of irritating and vesicant medications during PIT, no association was also observed in the crude Poisson regression model (RR 0.89; 95% CI 0.52-1.54; p-value = 0.69) and adjusted model for gender and age (RR 1.21; 95% CI 0.68-2.16; p-value = 0.53) compared to the difficulty in PICC insertion.

#### Indication of PICC use and insertion

The average time until PICC insertion in this study was 10.88 days (standard deviation 13.06), minimum zero and maximum 73 days and median of 6 days, considering the first day of hospitalization until the date of insertion of the PICC. The most frequently described indication for use was for the administration of antimicrobials (78.9%). The insertion site most used by nurses was the external jugular (54.6%). Regarding PICC use time, an average of 9.24 days (standard deviation 12.58) was detected, with a minimum of zero and a maximum of 78 days (median of 6 days).

More than half of the procedures occurred without difficulty in PICC insertion (56.0%). The average number of punctures for PICC insertion was 1.78 (standard deviation 1.02), at least one and maximum six. Ultrasonography was performed to assist with the insertion of 2 (2.6%) PICC. The success rate of the procedure was 97.6% among those who did not have difficulty inserting the PICC and 51.5% among those who had this difficulty.

Regarding PICC characteristics, 71 (97.2%) of the catheters were of the monolumen type, and 68 (93.1%) were of polyurethane composition, factors influenced by the purchase tender for that period.

 In the Poisson Regression ( Table 1 ), there was evidence of an association between the number of punctures and the difficulty in PICC insertion. Using the model adjusted for gender and age, it is estimated that, on average, with each increase of one puncture, the risk of difficulty increases by 42% (RR 1.42; 95% CI 1.11-1.81). There was no association between the insertion site (external jugular or upper limbs) and difficulty in PICC insertion (p=0.18). 

**Table 1 -:** Crude and adjusted logistic regression models of difficulty inserting the Peripherally Inserted Central Catheter (PICC), with relative risk calculation (n = 76). Londrina, PR, Brazil, 2020

**Comparison with the difficulty of PICC** * **insertion**	**Crude**				**Adjusted**			
	**Relative risk** ^†^	**Confidence interval (95%)**		**p** **Value**	**Relative risk** ^†^	**Confidence interval** **(95%)**		**p** **Value**
External Jugular vs. Upper limbs	0.48	0.28	0.84	<0.01	0.67	0.38	1.20	0.18
Number of Punctures (with each increase of one puncture)	1.57	1.29	1.92	<0.01	1.42	1.11	1.81	<0.01

* Peripherally Inserted Central Catheter

^†^ Model adjusted for gender and age ( *n* = 33)

#### Complications using PICC

 Complications are relevant in this study and were present in 72.8% of patients using PICC. There was no association between the presence of complications in the use of PICC and the time of PIT, both in the crude model (RR 0.99; 95% CI 0.97-1.00; p-value 0.08) and in the adjusted model (RR 0.99; 95% CI 0.98-1.00; p-value 0.08) ( Table 2 ). 

 In Table 3 it is possible to observe that secondary migration of the tip was the most cited (53.4%), followed by phlebitis (16.2%) and primary migration of the tip (11.6%). It is important to mention that, for Tables 3, 4 and 5, successful insertions were considered (n=59), since the descriptive analysis aimed to describe the complications of PICC use. 

 More than half of the patients had complications that led to PICC removal (54.2%), mostly due to secondary migration of the tip (62.5%), as shown in Table 4 . The average time, in days, between the presence of a complication and removal was 7.6 (standard deviation 12.8), minimum zero and maximum 73 days. The average complication-free time was 8.8 days. 

**Table 2:** - Crude and adjusted logistic regression models of the presence of complications in the use of the Peripherally Inserted Central Catheter (PICC), with relative risk calculation (n = 59). Londrina, PR, Brazil, 2020

**Comparison with the presence of complications in the use of PICC** *	**Crude**				**Adjusted**			
	**Relative risk** ^†^	**Confidence interval (95%)**		**p Value**	**Relative risk** ^†^	**Confidence interval (95%)**		**p Value**
Prior Intravenous Therapy Time: with each increase of one day	0.99	0.97	1.00	0.08	0.99	0.98	1.00	0.08
PICC* use time: with each increase of one day	1.01	1.0009	1.01	0.02	1.01	0.998	1.02	0.12

^*^ Peripherally Inserted Central Catheter

^†^ Adjusted model controlled by gender, age and hospitalization unit ( *n* = 43)

**Table 3 -:** Description of variables related to complications in the use of the Peripherally Inserted Central Catheter (PICC) in hospitalized adult patients (n = 59). Londrina, PR, Brazil, 2020

**Variables**	**n**	**%**
**Presence of complication**		
Yes	43	72.8
No	16	27.1
**Secondary migration**		
Yes	31	70.4
**Phlebitis**		
Yes	13	30.2
**Suspected infection**		
Yes	8	18.6
**Primary migration**		
Yes	6	13.9
**Total occlusion**		
Yes	5	11.6
**Bleeding**		
Yes	5	11.6
**Catheter damage**		
Yes	4	9.3
**Catheter-associated bloodstream infection**		
Yes	1	2.3
**Skin injury**		
Yes	1	2.3
**Venous thrombosis**		
Yes	0	0.0

**Table 4 -:** Description of variables related to the removal of the Peripherally Inserted Central Catheter (PICC) due to complications in hospitalized adult patients (n = 59). Londrina, PR, Brazil, 2020

**Variables**	**n**	**%**
**PICC** * **removal during hospitalization caused by complication**		
Yes	32	54.2
No	27	45.8
**Secondary migration**		
Yes	20	62.6
**Suspected infection**		
Yes	6	18.8
**Catheter damage**		
Yes	2	6.2
**Total occlusion**		
Yes	2	6.2
**Phlebitis**		
Yes	1	3.1
**Catheter-associated bloodstream infection**		
Yes	1	3.1

* Peripherally Inserted Central Catheter

For study participants who had some type of complication when using the PICC, the majority (78.3%) had the catheter inserted in the external jugular vein compared to the upper limbs. The average age at admission was higher (53.51 years) among patients who had complications using the PICC.

Table 2 shows the association in the crude Poisson model for the time of PICC use with each increase of one day in relation to the incidence of complications (RR 1.01; 95% CI 1.0009-1.01; p-value 0.02). 

 In the Poisson analysis with robust variance presented in Table 5 , there was evidence of an association between the PICC insertion site and its removal due to complications, with insertion in the external jugular presenting a higher risk of removal due to complications in relation to the upper limbs (RR 2.12; 95% CI 1.11-4.07; p-value 0.02). Considering the secondary migration outcome, there was no evidence of association with the insertion site. 

**Table 5 -:** Poisson regression model with robust variance and relative risk calculation (n = 59). Londrina, PR, Brazil, 2020

**Variable**	**Site**					
	**External Jugular**	**Upper limbs**	**Relative Risk** **(External Jugular vs Limbs)**	**Confidence interval (95%)**		**p Value**
PICC* removal due to complications (n=59)						
No	12 (32.4)	15 (68.2)				
Yes	25 (67.6)	7 (31.8)	2.12	1.11	4.07	0.02
Complication: secondary migration (n=43)						
No	6 (23.3)	6 (42.9)				
Yes	23 (76.7)	8 (57.1)	1.34	0.82	2.20	0.24

* Peripherally Inserted Central Catheter

## Discussion

 The approach of this study in associating PIT with PICC use and complications is original. Successive failures in establishing venous access via SPIVC lead to several puncture attempts that have multiple impacts, including financial ones ^(^ 7 ^)^ . 

 Preservation of peripheral veins during hospitalization is crucial to reduce complications, ensure safety and promote patient satisfaction ^(^ 15 ^)^ . However, it was observed that the number of peripheral punctures before PICC insertion negatively impacted first-attempt PICC insertion success rates ^(^ 12 ^,^ 16 ^-^ 18 ^)^ . It is believed that this finding may be related to the late indication of PICC, which contributes to the choice of non-recommended veins, such as the external jugular. 

 The infusion of vesicant and irritating medications through the PIVC also contributes to vascular fragility. Despite recurrent peripheral infusion, administering them in this way is contraindicated and associated with complications of PIVC ^(^ 18 ^-^ 19 ^)^ . In this study, no association was identified between the administration of these drugs and difficulty inserting the PICC. 

 The high frequency of PICC insertion in the external jugular vein was a particularity of this study, incongruous with the recommendations of national and international guidelines ^(^ 1 ^,^ 20 ^)^ . In the literature, PICC insertion into the external jugular vein was not identified. The first vein of choice for PICC puncture and insertion should be the basilica, and the veins above the antecubital fossa are the most appropriate for PICC insertion, a finding that appears in several studies, including national ones ^(^ 21 ^-^ 23 ^)^ . It is reiterated that insertion into the external jugular vein increased the risk of removal and difficulty in inserting the PICC more than once, compared to the upper limbs. 

 Traditionally, external jugular vein puncture has specific indications, including patients who do not have other more visible veins, for example in the upper limbs, or when vascular visualization technology is not available ^(^ 24 ^)^ . Anatomically, it is a more prominent and visible vein than the others ^(^ 24 ^)^ , and as ultrasound was used in only two punctures and the peripheral venous network was impaired, this was possibly the reason why the jugular vein became the only viable and preferred option for inserting catheters, in this case, the PICC. 

 There was no evidence of an association between the insertion site (external jugular or upper limbs) and difficulty in PICC insertion, as shown in Table 1 . However, it is considered that, due to anatomical factors and depletion of the peripheral venous network, the external jugular vein is more preserved. Furthermore, the nurse, with a view to the success of the procedure, may be more successful when inserting this vein, compared to the other veins in the upper limbs, which are in a fragile state and present greater insertion complications due to reduced visualization ^(^ 13 ^)^ . 

 Although used in this anatomical area, the fixation of PICC and other CVAD in external jugular veins has become a challenge that inspires diverse nursing care. Complications such as migration and fixation of the dressing can culminate in the removal of the PICC, since the neck is an area of great movement, diaphoresis and hair growth ^(^ 25 ^)^ . 

Table 5 showed that secondary migration was not associated with PICC insertion in the external jugular vein when compared to the upper limbs. However, still in Table 5 , the removal of the PICC caused by complications was associated with the insertion site in the external jugular, demonstrating the difficulty in PICC care in this region. 

 Several factors can contribute to the difficulties or success of the PICC insertion procedure. One of the most important is vascular fragility, which emphasizes the importance of preserving peripheral veins ^(^ 13 ^)^ . In this study, the external jugular was the most used insertion site. It is believed that, by favoring visualization, this vein facilitates puncture. 

 As an alternative to this difficult visualization and palpation of the vessels, the use of ultrasound becomes extremely effective in ensuring the success of the procedure and reducing complications ^(^ 1 ^)^ . However, in the present study, two insertions were guided by ultrasound. 

 Such vascular visualization technologies, in addition to providing greater success in the procedure by avoiding several unsuccessful puncture attempts, provide comfort in comparison with conventional direct puncture techniques ^(^ 26 ^)^ . It was identified that, by increasing the number of punctures, the risk of difficulty in insertion increased. 

 The indication for the use of PICC remains mainly for antibiotic therapy, corroborating the existing literature ^(^ 22 ^,^ 27 ^)^ . The average time of PICC use in this study proved to be appropriate in accordance with recommendations ^(^ 1 ^,^ 28 ^)^ . Research indicates a lower prevalence of complications when using PICC ^(^ 21 ^-^ 22 ^,^ 29 ^)^ , with an important finding in this study of secondary tip displacement. 

 Considering that complications related to PICC may result in the need to remove the device, one of the outcomes caused is the interruption and consequent delay of therapy. Primary or secondary migration in this study was much higher compared to previous research findings ^(^ 4 ^,^ 21 ^-^ 23 ^)^ . However, the hospital in question, like many others in the Brazilian scenario, did not have standardization of stabilization devices that reduce the risk of displacement, due to the high cost ^(^ 1 ^)^ . 

The convenience sample was a limiting factor in this study. Similar to other public hospitals in the country, bidding processes interfere with the availability of PICC and the modification of brands and composition of materials.

This study supports nurses’ understanding of PICC insertion, highlighting the importance of this professional’s knowledge about the preservation of the peripheral venous network prior to its insertion and adequate indication of the catheter, in addition to identifying complications in the use of this device. By emphasizing the importance of nurses’ clinical judgment, this study aims to contribute to optimizing the PICC insertion process and reducing associated complications.

## Conclusion

Discussions about the preservation of the peripheral venous network and indication of the appropriate catheter during PIT for hospitalized adult patient are still little explored in the literature. In this study, unfortunately, a high prevalence of administration of vesicant medications during PIT was identified, which can culminate in the depletion of the vascular network, but without a statistical association. Regarding PIT time, there was no association with the presence of complications in the use of PICC.

Secondary migration has been reported as the main complication during PICC use and the most prevalent cause of complication removal.

It is believed that the presence of weakened and compromised veins makes the PICC insertion procedure difficult, and may be related to the delay in PICC indication.

It was also found that the number of punctures for PICC insertion increases the difficulty of inserting it.

Contradictory to national and international findings, PICC insertion into the external jugular vein was recurrent. Furthermore, it was shown that insertion in the external jugular vein presents a greater risk of removal due to complications in relation to the upper limbs. There was no association between the insertion site (external jugular or upper limbs) and difficulty in PICC insertion.

The scarcity of studies relating the indication and use of PICC in adult patients highlights the importance of this research and the need to develop safe health practices, as well as publications on the topic in question.
