# Supplementary material for: Complications in the use of peripherally inserted central catheter associated with peripheral intravenous therapy: retrospective cohort
Source: Rev Lat Am Enfermagem. 2024 Sep 23;32:e4341. doi: 10.1590/1518-8345.7173.4341 (PMC11421515; doi:10.1590/1518-8345.7173.4341)
Supplement: Translation

# Translation: Complicaciones en el uso de catéter central de inserción periférica asociadas a la terapia intravenosa periférica: cohorte retrospectiva *

**DOI:** 10.1590/1518-8345.7173.4340

**Published:** 2024-09-23

**Authors:** Elizângela Santana dos Santos, Elaine Barros Ferreira, Fernanda Titareli Merizio Martins Braga, Amanda Salles Margatho, Paulo Sousa, Renata Cristina de Campos Pereira Silveira

**Affiliations:** 1 Universidade de São Paulo, Escola de Enfermagem de Ribeirão Preto, Centro Colaborador de la OPS/OMS para el Desarrollo de la Investigación en Enfermería, Ribeirão Preto, SP, Brasil; ^2^ Universidade de Brasília, Faculdade de Ciências da Saúde, Brasília, DF, Brasil; ^3^ Universidade Nova de Lisboa, Escola Nacional de Saúde Pública, Lisboa, Estremadura, Portugal

**Keywords:** Cateterismo Venoso Central, Infusiones Intravenosas, Enfermería, Cateterismo Periférico, Seguridad del Paciente, Atención de Enfermería

## Abstract

**Objetivo:**

analizar la aparición de dificultad en la inserción periférica del catéter central y la presencia de complicaciones en el uso de este dispositivo en adultos hospitalizados que recibieron terapia intravenosa periférica a través de un catéter intravenoso periférico corto e identificar si existe asociación entre la terapia intravenosa periférica y la presencia de complicaciones en el uso del catéter central de inserción periférica.

**Método:**

cohorte retrospectiva, con pacientes de 18 años o más, en un hospital universitario terciario, con catéter central de inserción periférica, que tuvieran al menos un catéter intravenoso periférico corto previo. Los datos se analizaron mediante estadística descriptiva y regresión de Poisson.

**Resultados:**

la muestra estuvo compuesta por 76 pacientes. Hubo asociación entre dificultad en el procedimiento de inserción y número de punciones (p<0,01) e inserción en la vena yugular externa en comparación con los miembros superiores (p<0,01). El sitio de inserción también se asoció con la retirada del catéter central insertado periféricamente debido a complicaciones en el análisis de varianza robusto (p=0,02). No se identificaron asociaciones entre: dificultad para insertar el dispositivo y tiempo de terapia intravenosa periférica (modelo crudo p=0,23; modelo ajustado p=0,21); dificultad en la inserción y administración de medicación irritante y vesicante (modelo crudo p=0,69; modelo ajustado p=0,53); complicación en el uso de catéter central de inserción periférica y tiempo de terapia intravenosa periférica (modelos crudo y ajustado p=0,08); y migración secundaria de la punta del catéter y el sitio de inserción del dispositivo (p=0,24).

**Conclusión:**

se pudo identificar la migración secundaria como una de las principales complicaciones, resultando en la retirada prematura del dispositivo. Además, cuanto mayor sea el número de intentos de punción para insertar el PICC, mayor será la dificultad para insertarlo. La inserción en vena yugular externa fue recurrente, con mayor riesgo de extracción por complicaciones en relación a los miembros superiores.

## Destacados

**(1)** No hubo asociación entre tiempo de TIP y dificultad para insertar el PICC.

**(2)** No hubo asociación entre tiempo de TIP y complicación en el uso del PICC.

**(3)** La migración secundaria de la punta del PICC fue la complicación mencionada con mayor frecuencia.

**(4)** La vena yugular externa se asoció con la dificultad en la inserción del PICC.

**(5)** La vena yugular externa se asoció con la presencia de complicaciones del PICC.

## Introducción

 La terapia de infusión consiste en la infusión de líquidos por vía vascular, intraósea, subcutánea o intratecal. Específicamente para pacientes que reciben terapia nutricional, componentes sanguíneos o medicamentos por vía intravenosa o vascular, se denomina terapia intravenosa ^(^ 1 ^)^ . 

 Para establecer la Terapia Intravenosa Periférica (TIP), se utilizan Catéteres Intravenosos Periféricos (CIVP). De estos, el Catéter Intravenoso Periférico Corto (CIVPC) es el más utilizado en pacientes hospitalizados ^(^ 1 ^)^ . 

 Se estima que el 80% de los pacientes hospitalizados utilizan CIVPC ^(^ 2 ^)^ . Sin embargo, a pesar de los beneficios e indicaciones, existen complicaciones asociadas al uso de este dispositivo. Estas complicaciones pueden clasificarse como locales o sistémicas, como flebitis ^(^ 3 ^)^ , migración primaria y secundaria de la punta ^(^ 4 ^)^ , Trombosis Venosa Profunda (TVP) ^(^ 4 ^)^ , daño del catéter, oclusión ^(^ 3 ^)^ e Infección del Torrente Sanguíneo Asociada a Catéter (ITSAC) ^(^ 1 ^,^ 5 ^)^ . 

 Para pacientes que requieren administración de medicamentos incompatibles de forma periférica, terapia intravenosa por un período de tiempo prolongado o sin condiciones para punción periférica, puede estar indicado el uso de un Dispositivo de Acceso Vascular Central (DAVC), uno de los cuales es el *Peripherally Inserted Central Catheter* (PICC) ^(^ 1 ^)^ . 

 El PICC es un catéter largo, flexible, que tiene indicaciones específicas de uso, como terapia con antibióticos por más de seis días, uso de medicamentos irritantes o vesicantes, pH extremos y osmolaridad alta. Se inserta por medio de una vena periférica y logra un posicionamiento central en el tercio distal de la unión cavo-auricular ^(^ 1 ^)^ . 

 En Brasil, el PICC es insertado mayoritariamente por enfermeros debidamente calificados, y esta práctica está legalizada por la Resolución nº 258 del *Conselho Federal de Enfermagem* (COFEN) ^(^ 6 ^)^ . Sin embargo, el uso y adquisición de este dispositivo en hospitales del *Sistema Único de Saúde* (SUS) aún son limitados, debido al alto costo y a aspectos culturales que incluyen desconocimiento del catéter y recomendaciones de algunos profesionales ^(^ 7 ^)^ . 

 El uso del PICC está descrito en la literatura, principalmente, para población pediátrica y neonatal ^(^ 8 ^-^ 11 ^)^ . A pesar de ser considerado un catéter de primera elección, muchas veces enfrenta retrasos en su inserción, al no ser insertado en el momento correcto, lo que provoca daños importantes y agotamiento de la red venosa periférica en pacientes hospitalizados ^(^ 12 ^)^ . 

 En este escenario desafiante, es importante comprender las dificultades que se enfrentan al insertar el PICC y las posibles complicaciones asociadas con el uso de este dispositivo. Se trata de un conocimiento importante, especialmente porque el enfermero, ante la necesidad de insertarlo para garantizar una terapia intravenosa segura y eficaz, puede encontrarse con la limitación de las condiciones de las venas periféricas como factor que impide una adecuada inserción ^(^ 1 ^,^ 13 ^)^ . 

Además, comprender los factores que contribuyen a una inserción fallida o la incidencia de complicaciones relacionadas con el uso del PICC puede ayudar al equipo de salud a actuar para reducir estas complicaciones. Esto, a su vez, mejora la calidad de la atención brindada y favorece la seguridad del paciente adulto que utiliza PICC, ya que la investigación con esta población es escasa. Por lo tanto, el objetivo de este estudio fue analizar la aparición de dificultad en la inserción periférica del catéter central y la presencia de complicaciones en el uso de este dispositivo en pacientes adultos hospitalizados que recibieron terapia intravenosa periférica previa a través de un catéter intravenoso periférico corto, así como identificar si existe asociación entre la terapia intravenosa periférica y la presencia de complicaciones al utilizar un catéter central de inserción periférica.

## Método

### Diseño del estudio

 Estudio de cohorte retrospectivo y longitudinal. Para informar la investigación, se utilizaron las recomendaciones del *Strengthening the Reporting of Observational Studies in Epidemiology* (STROBE). 

### Lugar

El estudio se realizó en un hospital de educación pública terciaria de la ciudad de Londrina, PR, Brasil, que cuenta con un comité de enfermeros capacitados y especializados en la inserción de PICC. Este comité sigue los protocolos institucionales, pero la elección de la vena para la inserción la realiza el enfermero que efectúa el procedimiento. No hubo capacitación para los enfermeros de la comisión durante el período del estudio.

### Período

Los datos fueron recolectados del 1 de enero al 31 de diciembre de 2020.

### Población

Adultos hospitalizados durante el período de recolección de datos que se sometieron a un procedimiento de inserción de PICC, que habían recibido terapia intravenosa periférica previa a través de un catéter intravenoso periférico corto.

### Criterios de selección

Se incluyeron todos los pacientes de 18 años o más, hospitalizados entre el 1 de enero y el 31 de diciembre de 2020, a quienes se les insertó con éxito o sin éxito un PICC y precedido por al menos un CIVPC. Se excluyeron aquellos con inserción previa de un DAVC, así como aquellos que fueron dados de alta del hospital con el PICC para uso domiciliario.

### Definición de la muestra

La muestra se definió por conveniencia.

### Variables del estudio

#### Variable dependiente

*Aparición de complicaciones durante el uso del PICC:* aparición de al menos un evento adverso durante el uso del PICC que no resultó en la extracción del dispositivo (migración primaria o secundaria, flebitis, sospecha de infección, oclusión total, daño del catéter, ITSAC, trombosis venosa, sangrado o lesión de la piel) ^(^ 1 ^)^ . 

#### Variables independientes

*Tiempo de TIP (variable principal):* duración, en días, desde el primer día de uso del CIVPC por parte del paciente hospitalizado hasta la fecha de inserción del PICC; 

*Migración primaria:* situación en la que la punta del PICC no está colocada en la vena cava superior o en la unión cavo-auricular ^(^ 1 ^)^ ; 

*Migración secundaria:* también llamada desplazamiento, es la salida total de la punta del PICC de la vena ^(^ 1 ^)^ ; 

*Flebitis:* aparición de uno o más síntomas (dolor y/o sensibilidad en el lugar de inserción y en el trayecto del PICC, eritema, edema, induración, drenaje purulento o cordón venoso palpable) ^(^ 1 ^)^ ; 

*Sospecha de infección:* síntomas locales o sistémicos de infección atribuidos al PICC sin confirmación de ITSAC ^(^ 1 ^)^ ; 

*Oclusión total:* uno o más lúmenes del catéter presentan obstrucción intraluminal, imposibilitando la inyección de líquidos o aspiración de sangre ^(^ 1 ^)^ ; 

*Daño del catéter:* signos de catéter visiblemente fracturado, fuga local, disfunción del catéter, evidencia radiográfica de extravasación o infiltración de tejido ^(^ 1 ^)^ ; 

*ITSAC:* infección primaria del torrente sanguíneo en un paciente que tenía un dispositivo de acceso central dentro de las 48 horas anteriores al desarrollo de la infección del torrente sanguíneo sin estar relacionada con una infección en otro lugar ^(^ 1 ^)^ ; 

*Trombosis venosa:* en este estudio se consideró la TVP, definida por la aparición de trombos visualizados mediante pruebas de imagen en el lugar del trayecto del PICC en un paciente con síntomas de dolor en el miembro cateterizado, edema o enrojecimiento ^(^ 1 ^)^ ; 

*Sangrado al insertar:* presencia de sangre visible en el sitio de inserción del dispositivo ^(^ 1 ^)^ ; 

*Lesión en la piel:* presencia de uno o más síntomas (enrojecimiento durante más de 30 minutos después de retirar el apósito, laceración de la piel, descamación de la piel, ampollas en la piel, lesiones traumáticas, pústula, vesícula, pápula o rotura de la piel) ^(^ 1 ^)^ ; 

*Tiempo de uso del PICC:* se considera el tiempo de uso del PICC, duración en días desde la inserción del dispositivo hasta su extracción; 

*Dificultad para insertar el PICC:* descripción por parte del enfermero que realizó la inserción del PICC en el expediente médico o en un formulario específico sobre la presencia de dificultades en el procedimiento de inserción; 

*Número de punciones:* número de punciones en el procedimiento de inserción del PICC; 

*Retirada del PICC:* último día de uso del PICC, fecha de extracción; 

*Unidad de internación:* lugar de ingreso hospitalario en el momento de la inserción del PICC (sala de emergencias, Unidad de Cuidados Intensivos (UCI) para adultos, unidad médico quirúrgica, centro quirúrgico, centro de tratamiento de quemaduras y UCI para quemados); 

*Punciones venosas periféricas previas al PICC* : cantidad de CIVPC durante la TIP; 

*Medicamentos vesicantes e irritantes utilizados en la TIP* : se considera medicamento vesicante aquel capaz de provocar ampollas, descamación o necrosis cuando hay extravasación ^(^ 1 ^)^ ; 

*Sitio de inserción del PICC:* sitio de inserción del PICC, las venas basílica, cefálica, axilar u otras se clasificaron como miembros superiores, y yugular externa; 

*Retirada del PICC causada por una complicación* : migración primaria o secundaria, flebitis, sospecha de infección, daño del catéter, oclusión total, ITSAC, TVP, sangrado o lesión de la piel que culminaron con la extracción del PICC. 

### Instrumentos utilizados para recolectar la información

Se utilizó un formulario elaborado por el grupo de investigación del autor, con información sobre TIP, condiciones de la red venosa, múltiples punciones periféricas, uso de medicamentos vesicantes y caracterización del paciente para recolectar datos de las historias clínicas. Además de esto, se utilizó un formulario de procedimiento de inserción del PICC, con información sobre la indicación de inserción del dispositivo, datos del PICC (material, calibre, lúmenes y longitud), sitio de inserción, vaso accedido, condiciones de la red venosa que dificultan la punción del vaso y la introducción del catéter, dificultad de inserción, radiografía de control y registro de uso de ecografía y datos sobre el retiro (motivo de retiro, integridad del catéter, cultivos de punta de catéter y hemocultivo).

### Recolección de datos

En la recolección de datos, se consultaron las historias clínicas de los pacientes y los formularios del procedimiento de inserción del PICC de los hospitalizados del 1 de enero al 31 de diciembre de 2020. Los formularios del procedimiento de inserción del PICC fueron completados por enfermeros del comité de terapia de infusión del hospital.

### Procesamiento y análisis de los datos

 Inicialmente, los datos se describieron mediante frecuencias absolutas y porcentajes (variables cualitativas) y mediante medidas como media, desviación estándar, mínimo, mediana y máximo (variables cuantitativas). Para estimar el Riesgo Relativo para los resultados de dificultad en la inserción, complicaciones durante la hospitalización, desplazamiento y retiro del PICC en relación a las variables de interés, se utilizó el modelo de regresión de Poisson con varianza robusta simple y múltiple (cuando correspondía). Se aplicó la prueba Log-rank para verificar la evidencia de diferencias entre las curvas de supervivencia. Los análisis se realizaron utilizando el *software* SAS 9.4. En este trabajo se adoptó un nivel de significancia del 5% (p < 0,05) y se aplicaron las pruebas de Kolmogorov-Smirnov o Shapiro-Wilk para normalidad y homocedasticidad. 

### Aspectos éticos

 El estudio fue presentado y aprobado por el Comité de Ética en Investigación (CEP, por sus siglas em portugués) de la *Escola de Enfermagem de Ribeirão Preto* (EERP) y por el CEP del *Hospital Universitário de Londrina* (HUL), bajo dictamen nº 3.627.165 y CAAE 16451019.8.0000.5393, según Resolución nº 466/2012 ^(^ 14 ^)^ . Se solicitó y obtuvo la renuncia al Formulario de Consentimiento Libre e Informado. 

## Resultados

### Participantes

De los 105 pacientes adultos hospitalizados a los que se les colocó un PICC, 28 fueron excluidos porque no tenían al menos un CIVPC antes del PICC y otro fue dado de alta para recibir tratamiento domiciliario con el dispositivo. La muestra final estuvo compuesta por 76 pacientes, de los cuales a 59 (77,6%) se les realizó la inserción del PICC con éxito y a 17 (22,3%) sin éxito.

La edad promedio de ingreso fue de 50 años (desviación estándar 18,29), mínima de 18 y máxima de 90 años (mediana de 47 años), mientras que el género más prevalente fue el masculino (69,74%).

En cuanto a las unidades de hospitalización, más de la mitad (51,3%) se encontraba en unidad de clínica médico-quirúrgica. Una particularidad de este estudio es la hospitalización en la UCI de Quemados, que alcanzó el 15,8%. Luego, el 21,0% de los pacientes ingresaron en la UCI de adultos.

#### Terapia intravenosa periférica antes del PICC

El tiempo medio en días que los pacientes utilizaron CIVPC fue de 10,53 (desviación estándar 31,58), con una mediana de 4 días y un máximo de tiempo de 274 días. El número medio de punciones venosas previas al PICC fue de 4,22 (desviación estándar 3,74), mediana de tres, al menos una y máximo de 8.

También se analizó el uso de medicamentos irritantes y vesicantes durante este período, revelando que el 72,3% de los participantes utilizaron este tipo de medicación. Los más recurrentes fueron: Cloruro de Potasio (48,2%), Clorhidrato de Vancomicina (29,0%) y Piperacilina sódica + Tazobactam sódico (25,4%).

No hubo asociación entre el tiempo de TIP (con cada aumento de un día) en comparación con la dificultad en la inserción del PICC en los modelos crudo (RR 0,99; IC 95% 0,98-1,00; valor p = 0,23) y ajustado por género y edad (RR 1,00; IC 95%: 0,99-1,00; valor p = 0,21).

Para la administración de medicamentos irritantes y vesicantes durante la TIP, tampoco se observó asociación en el modelo de regresión de Poisson crudo (RR 0,89; IC 95% 0,52-1,54; valor p = 0,69) y ajustado por género y edad (RR 1,21; 95 % IC 95% 0,68-2,16; valor p = 0,53) en comparación con la dificultad en la inserción del PICC.

#### Indicación de uso e inserción del PICC

El tiempo promedio hasta la inserción del PICC en este estudio fue de 10,88 días (desviación estándar 13,06), mínimo cero y máximo 73 días y mediana de 6 días, considerando el primer día de internación hasta la fecha de inserción del PICC. La indicación de uso descrita con mayor frecuencia fue la administración de antimicrobianos (78,9%). El sitio de inserción más utilizado por los enfermeros fue la yugular externa (54,6%). En cuanto al tiempo de uso del PICC, se detectó un promedio de 9,24 días (desviación estándar 12,58), con un mínimo de cero y un máximo de 78 días (mediana de 6 días).

Más de la mitad de los procedimientos transcurrieron sin dificultad en la inserción del PICC (56,0%). El número medio de punciones para la inserción del PICC fue de 1,78 (desviación estándar 1,02), mínimo uno y máximo seis. Se realizó una ecografía para ayudar con la inserción de 2 PICC (2,6%). La tasa de éxito del procedimiento fue del 97,6% entre quienes no tuvieron dificultad para insertar el PICC y del 51,5% entre quienes tuvieron esta dificultad.

En cuanto a las características del PICC, 71 (97,2%) de los catéteres eran del tipo monolumen y 68 (93,1%) eran de composición de poliuretano, factores influenciados por la licitación de compra para ese período.

 En la Regresión de Poisson ( Tabla 1 ), se evidenció asociación entre el número de punciones y la dificultad en la inserción del PICC. Utilizando el modelo ajustado por género y edad, se estima que, en promedio, con cada aumento de una punción, el riesgo de dificultad aumenta un 42% (RR 1,42; IC 95% 1,11-1,81). No hubo asociación entre el sitio de inserción (yugular externa o miembros superiores) y la dificultad en la inserción del PICC (p=0,18). 

**Tabla 1 -:** Modelos crudo y ajustado de regresión logística de la dificultad para insertar el *Peripherally Inserted Central Catheter* (PICC), con cálculo de riesgo relativo (n = 76). Londrina, PR, Brasil, 2020

**Comparación con la dificultad de inserción del PICC** *	**Crudo**				**Ajustado**			
	**Riesgo relativo** ^†^	**Intervalo de confianza (95%)**		**Valor p**	**Riesgo relativo** ^†^	**Intervalo de confianza (95%)**		**Valor p**
Yugular externa vs. Miembros superiores	0,48	0,28	0,84	<0,01	0,67	0,38	1,20	0,18
Número de Punciones (con cada aumento de una punción)	1,57	1,29	1,92	<0,01	1,42	1,11	1,81	<0,01

* *Peripherally Inserted Central Catheter*

^†^ Modelo ajustado por género y edad (*n*= 33)

#### Complicaciones en el uso del PICC

 Las complicaciones son relevantes en este estudio y estuvieron presentes en el 72,8% de los pacientes que utilizaron PICC. No hubo asociación entre la presencia de complicaciones en el uso del PICC y el tiempo de TIP, tanto en el modelo crudo (RR 0,99; IC 95% 0,97-1,00; valor p 0,08) como en el modelo ajustado (RR 0,99; IC 95% 0,98-1,00; valor p 0,08) ( Tabla 2 ). 

 En la Tabla 3 se puede observar que la migración secundaria de la punta fue la más mencionada (53,4%), seguida de la flebitis (16,2%) y la migración primaria de la punta (11,6%). Es importante mencionar que, para las Tablas 3, 4 y 5, se consideraron las inserciones exitosas (n=59), ya que el análisis descriptivo tuvo como objetivo describir las complicaciones del uso del PICC. 

 Más de la mitad de los pacientes tuvieron complicaciones que llevaron a la retirada del PICC (54,2%), principalmente debido a la migración secundaria de la punta (62,5%), como se muestra en la Tabla 4 . El tiempo promedio, en días, entre la presencia de una complicación y su extracción fue de 7,6 (desviación estándar 12,8), mínimo cero y máximo 73 días. El tiempo medio libre de complicaciones fue de 8,8 días. 

**Tabla 2 -:** Modelos crudo y ajustado de regresión logística de la presencia de complicaciones en el uso del *Peripherally Inserted Central Catheter* (PICC), con cálculo de riesgo relativo (n = 59). Londrina, PR, Brasil, 2020

**Comparación con la presencia de complicaciones en el uso del PICC** *	**Crudo**				**Ajustado**			
	**Riesgo relativo** ^†^	**Intervalo de confianza (95%)**		**Valor p**	**Riesgo relativo** ^†^	**Intervalo de confianza (95%)**		**Valor p**
Tiempo de Terapia Intravenosa Previa: con cada aumento de un día	0,99	0,97	1,00	0,08	0,99	0,98	1,00	0,08
Tiempo de uso del PICC*: con cada aumento de un día	1,01	1,0009	1,01	0,02	1,01	0,998	1,02	0,12

^*^ *Peripherally Inserted Central Catheter*

^†^ Modelo ajustado controlado por género, edad y unidad de internación (*n*= 43)

**Tabla 3 -:** Descripción de las variables relacionadas con complicaciones en el uso del *Peripherally Inserted Central Catheter* (PICC) en pacientes adultos hospitalizados (n = 59). Londrina, PR, Brasil, 2020

**Variables**	**n**	**%**
**Presencia de complicación**		
Sí	43	72,8
No	16	27,1
**Migración secundaria**		
Sí	31	70,4
**Flebitis**		
Sí	13	30,2
**Sospecha de infección**		
Sí	8	18,6
**Migración primaria**		
Sí	6	13,9
**Oclusión total**		
Sí	5	11,6
**Sangrado**		
Sí	5	11,6
**Daño del catéter**		
Sí	4	9,3
**Infección del torrente sanguíneo asociada a catéter**		
Sí	1	2,3
**Lesión en la piel**		
Sí	1	2,3
**Trombosis venosa**		
Sí	0	0,0

**Tabla 4 -:** Descripción de las variables relacionadas a la retirada por complicación del *Peripherally Inserted Central Catheter* (PICC) en pacientes adultos hospitalizados (n = 59). Londrina, PR, Brasil, 2020

**Variables**	**n**	**%**
**Retirada del PICC** [Table-fn tfn05]* **durante la hospitalización causada por complicación**		
Sí	32	54,2
No	27	45,8
**Migración secundaria**		
Sí	20	62,6
**Sospecha de infección**		
Sí	6	18,8
**Daño del catéter**		
Sí	2	6,2
**Oclusión total**		
Sí	2	6,2
**Flebitis**		
Sí	1	3,1
**Infección del torrente sanguíneo asociada a catéter**		
Sí	1	3,1

* *Peripherally Inserted Central Catheter*

Para los participantes del estudio que tuvieron algún tipo de complicación al utilizar el PICC, a la mayoría (78,3%) se les insertó el catéter en la vena yugular externa, en comparación con los miembros superiores. La edad media de ingreso fue mayor (53,51 años) entre los pacientes que tuvieron complicaciones con el uso del PICC.

 En la Tabla 2 se muestra la asociación en el modelo crudo de Poisson para el tiempo de uso del PICC con cada aumento de un día en relación a la incidencia de complicación (RR 1,01; IC 95% 1,0009-1,01; valor p 0,02). 

 En el análisis de Poisson con varianza robusta presentado en la Tabla 5 , hubo evidencia de una asociación entre el sitio de inserción del PICC y su extracción por complicaciones, presentando la inserción en la yugular externa un mayor riesgo de extracción por complicaciones en relación a los miembros superiores (RR 2,12; IC 95% 1,11-4,07; valor p 0,02). Considerando el resultado de migración secundaria, no hubo evidencia de asociación con el sitio de inserción. 

**Tabla 5 -:** Modelo de regresión de Poisson con varianza robusta y cálculo de riesgo relativo (n = 59). Londrina, PR, Brasil, 2020

**Variable**	**Sitio**					
	**Yugular Externa**	**Miembros Superiores**	**Riesgo Relativo** **(Yugular Externa** vs **Miembros)**	**Intervalo de confianza (95%)**		**Valor p**
Retirada del PICC* por complicaciones (n=59)						
No	12 (32,4)	15 (68,2)				
Sí	25 (67,6)	7 (31,8)	2,12	1,11	4,07	0,02
Complicación: migración secundaria (n=43)						
No	6 (23,3)	6 (42,9)				
Sí	23 (76,7)	8 (57,1)	1,34	0,82	2,20	0,24

^*^ *Peripherally Inserted Central Catheter*

## Discusión

 El enfoque de este estudio al asociar la TIP con el uso del PICC y sus complicaciones es original. Los sucesivos fracasos en el establecimiento del acceso venoso a través de CIVPC dan lugar a varios intentos de punción que tienen múltiples impactos, incluido el financiero ^(^ 7 ^)^ . 

 La preservación de las venas periféricas durante la hospitalización es crucial para reducir las complicaciones, garantizar la seguridad y promover la satisfacción de los pacientes ^(^ 15 ^)^ . Sin embargo, se observó que el número de punciones periféricas antes de la inserción del PICC tuvo un impacto negativo en las tasas de éxito de la inserción del PICC en el primer intento ^(^ 12 ^,^ 16 ^-^ 18 ^)^ . Se cree que este hallazgo puede estar relacionado con la indicación tardía del PICC, lo que contribuye a la elección de venas no recomendadas, como la yugular externa. 

 La infusión de medicamentos vesicantes e irritantes a través del CIVP también contribuye a la fragilidad vascular. A pesar de la infusión periférica recurrente, administrarlos de esta manera está contraindicado y asociado con complicaciones de CIVP ^(^ 18 ^-^ 19 ^)^ . En este estudio no se identificó asociación entre la administración de estos fármacos y la dificultad para insertar el PICC. 

 La alta frecuencia de inserción del PICC en vena yugular externa fue una particularidad de este estudio, incongruente con las recomendaciones de *guidelines* nacionales e internacionales ^(^ 1 ^,^ 20 ^)^ . En la literatura no se identificó la inserción del PICC en vena yugular externa. La primera vena de elección para la punción e inserción del PICC debe ser la basílica, siendo las venas por encima de la fosa antecubital las más apropiadas para la inserción del PICC, hallazgo encontrado en varios estudios, incluidos los nacionales ^(^ 21 ^-^ 23 ^)^ . Se reitera que la inserción en la vena yugular externa aumentó el riesgo de extracción y dificultad para insertar el PICC más de una vez, en comparación con los miembros superiores. 

 Tradicionalmente, la punción de la vena yugular externa tiene indicaciones específicas, incluidos pacientes que no tienen otras venas más evidentes, por ejemplo en los miembros superiores, o cuando no se dispone de tecnología de visualización vascular ^(^ 24 ^)^ . Anatómicamente, es una vena más prominente y visible que las demás ^(^ 24 ^)^ , y ya que solamente en dos punciones se utilizó la ecografía y la red venosa periférica se vio afectada, posiblemente esta fue la razón por la que la vena yugular pasó a ser la única vena viable y opción preferida para la inserción de catéteres, en este caso, el PICC. 

 No hubo evidencia de asociación entre el sitio de inserción (yugular externa o miembros superiores) y la dificultad en la inserción del PICC, como se muestra en la Tabla 1 . Sin embargo, se considera que, debido a factores anatómicos y al agotamiento de la red venosa periférica, la vena yugular externa esté más conservada. Además, el enfermero, de cara al éxito del procedimiento, tendrá más posibilidad de lograrlo al insertar esta vena, en comparación con las demás venas de los miembros superiores, que se encuentran en estado frágil y presentan mayores complicaciones de inserción por reducción de la visualización ^(^ 13 ^)^ . 

 Aunque se utiliza en esta área anatómica, la fijación del PICC y otros DAVC en venas yugulares externas se ha convertido en un desafío que inspira diversos cuidados de enfermería. Complicaciones como migración y fijación del apósito pueden culminar con la retirada del PICC, ya que el cuello es una zona de gran movimiento, diaforesis y crecimiento de pelos ^(^ 25 ^)^ . 

 La Tabla 5 mostró que la migración secundaria no se asoció con la inserción del PICC en vena yugular externa en comparación con los miembros superiores. Sin embargo, todavía en la Tabla 5 , la extracción del PICC causada por complicaciones se asoció con el sitio de inserción en yugular externa, lo que demuestra la dificultad del cuidado del PICC en esta región. 

 Varios factores pueden contribuir a las dificultades o al éxito del procedimiento de inserción del PICC. Uno de los más importantes es la fragilidad vascular, que destaca la importancia de preservar las venas periféricas ^(^ 13 ^)^ . En este estudio, la yugular externa fue el sitio de inserción más utilizado. Se cree que, al favorecer la visualización, esta vena facilita la punción. 

 Como alternativa a esta difícil visualización y palpación de los vasos, el uso de la ecografía se vuelve sumamente eficaz para garantizar el éxito del procedimiento y reducir las complicaciones ^(^ 1 ^)^ . Sin embargo, en el presente estudio, dos inserciones fueron guiadas por ecografía. 

 Estas tecnologías de visualización vascular, además de proporcionar un mayor éxito en el procedimiento al evitar varios intentos fallidos de punción, aportan comodidad en comparación con las técnicas convencionales de punción directa ^(^ 26 ^)^ . Se identificó que, al aumentar el número de punciones, se ampliaba el riesgo de dificultad en la inserción. 

 La indicación para el uso del PICC sigue siendo principalmente para la terapia con antibióticos, corroborando la literatura existente ^(^ 22 ^,^ 27 ^)^ . El tiempo promedio de uso del PICC en este estudio resultó ser apropiado, de acuerdo con las recomendaciones ^(^ 1 ^,^ 28 ^)^ . Las investigaciones indican una menor prevalencia de complicaciones al utilizar PICC ^(^ 21 ^-^ 22 ^,^ 29 ^)^ , con un hallazgo importante en este estudio del desplazamiento secundario de la punta. 

 Considerando que las complicaciones relacionadas con el PICC pueden derivar en la necesidad de retirar el dispositivo, uno de los resultados provocados es la interrupción y consecuente retraso de la terapia. La migración primaria o secundaria en este estudio fue mucho mayor en relación a los hallazgos de investigaciones anteriores ^(^ 4 ^,^ 21 ^-^ 23 ^)^ . Sin embargo, el hospital en cuestión, como muchos otros en el escenario brasileño, no contaba con estandarización de dispositivos de estabilización que reducen el riesgo de desplazamiento, debido al alto costo ^(^ 1 ^)^ . 

La muestra por conveniencia fue un factor limitante en este estudio. Al igual que en otros hospitales públicos del país, los procesos de licitación interfieren con la disponibilidad de PICC y la modificación de marcas y composición de materiales.

Este estudio apoya la comprensión del enfermero sobre la inserción del PICC, destacando la importancia del conocimiento de este profesional sobre la preservación de la red venosa periférica antes de su inserción y adecuada indicación del catéter, además de identificar complicaciones en el uso de este dispositivo. Al enfatizar la importancia del juicio clínico del enfermero, este estudio pretende contribuir a optimizar el proceso de inserción del PICC y reducir las complicaciones asociadas.

## Conclusión

Las discusiones sobre la preservación de la red venosa periférica y la indicación del catéter apropiado durante la TPI para paciente adulto hospitalizado aún están poco exploradas en la literatura. Desafortunadamente, en este estudio se identificó una alta prevalencia de administración de medicamentos vesicantes durante la TIP, que puede culminar en el agotamiento de la red vascular, pero sin asociación estadística. En cuanto al tiempo TIP, no hubo asociación con la presencia de complicaciones en el uso del PICC.

Se ha informado que la migración secundaria es la principal complicación durante el uso del PICC y la causa más frecuente de extracción por complicaciones.

Se cree que la presencia de venas debilitadas y comprometidas dificulta el procedimiento de inserción del PICC y puede estar relacionada con el retraso en la indicación del PICC.

También se encontró que el número de punciones para la inserción del PICC aumenta la dificultad de su inserción.

Contrariamente a los hallazgos nacionales e internacionales, la inserción del PICC en vena yugular externa fue recurrente. Además, se demostró que la inserción en la vena yugular externa presenta mayor riesgo de extracción por complicaciones en relación a los miembros superiores. No hubo asociación entre el sitio de inserción (yugular externa o miembros superiores) y la dificultad en la inserción del PICC.

La escasez de estudios que relacionen la indicación y uso del PICC en pacientes adultos resalta la importancia de esta investigación y la necesidad de desarrollar prácticas de salud seguras, así como publicaciones sobre el tema en cuestión.

# Translation: Complicações no uso do cateter central inserido perifericamente associadas à terapia intravenosa periférica: coorte retrospectiva *

**DOI:** 10.1590/1518-8345.7173.4342

**Published:** 2024-09-23

**Authors:** Elizângela Santana dos Santos, Elaine Barros Ferreira, Fernanda Titareli Merizio Martins Braga, Amanda Salles Margatho, Paulo Sousa, Renata Cristina de Campos Pereira Silveira

**Affiliations:** 1 Universidade de São Paulo, Escola de Enfermagem de Ribeirão Preto, Centro Colaborador de la OPS/OMS para el Desarrollo de la Investigación en Enfermería, Ribeirão Preto, SP, Brasil; ^2^ Universidade de Brasília, Faculdade de Ciências da Saúde, Brasília, DF, Brasil; ^3^ Universidade Nova de Lisboa, Escola Nacional de Saúde Pública, Lisboa, Estremadura, Portugal

**Keywords:** Cateterismo Venoso Central, Infusões Intravenosas, Enfermagem, Cateterismo Periférico, Segurança do Paciente, Cuidados de Enfermagem

## Abstract

**Objetivo:**

analisar a ocorrência de dificuldade na inserção periférica do cateter central e a presença de complicações no uso deste dispositivo em adultos hospitalizados que receberam terapia intravenosa periférica por cateter intravenoso periférico curto e identificar se há associação entre terapia intravenosa periférica e presença de complicações no uso do cateter central inserido perifericamente.

**Método:**

coorte retrospectiva, com pacientes de idade igual ou superior a 18 anos, em hospital terciário de ensino, com cateter central inserido perifericamente, que tiveram ao menos um cateter intravenoso periférico curto prévio. Os dados foram analisados por meio de estatística descritiva e regressão de Poisson.

**Resultados:**

a amostra foi constituída por 76 pacientes. Houve associação entre dificuldade no procedimento de inserção e número de punções (p<0,01) e inserção em veia jugular externa em comparação aos membros superiores (p<0,01). O local de inserção também apresentou associação com a remoção do cateter central inserido perifericamente por complicações na análise de variância robusta (p=0,02). Não foram identificadas associações entre: dificuldade de inserção do dispositivo com tempo de terapia intravenosa periférica (modelo bruto p=0,23; modelo ajustado p=0,21); dificuldade de inserção com administração de medicamento irritante e vesicante (modelo bruto p=0,69; modelo ajustado p=0,53); complicação no uso do cateter central inserido perifericamente e tempo de terapia intravenosa periférica (modelo bruto e ajustado p=0,08); e migração secundária da ponta do cateter com local de inserção do dispositivo (p=0,24).

**Conclusão:**

foi possível identificar a migração secundária como uma das principais complicações, resultando na remoção prematura do dispositivo. Além disso, quanto maior o número de tentativas de punções para a inserção do PICC, maior a dificuldade de inseri-lo. A inserção em veia jugular externa foi recorrente, sendo evidenciado maior risco de remoção por complicação em relação aos membros superiores.

## Destaques

**(1)** Não houve associação entre tempo de TIP e dificuldade na inserção do PICC.

**(2)** Não houve associação entre tempo de TIP e complicação no uso do PICC.

**(3)** Migração de ponta secundária do PICC foi a complicação mais referida.

**(4)** A veia jugular externa esteve associada à dificuldade na inserção do PICC.

**(5)** A veia jugular externa esteve associada à presença de complicações do PICC.

## Introdução

 A terapia infusional consiste na infusão de fluidos por via vascular, intraóssea, subcutânea ou intratecal. Especificamente para os pacientes que recebem a terapêutica nutricional, hemocomponentes ou medicamentos por via endovenosa ou vascular, é denominada terapia intravenosa ^(^ 1 ^)^ . 

 Para estabelecer a Terapia Intravenosa Periférica (TIP), são empregados os Cateteres Intravenosos Periféricos (CIVP). Destes, o Cateter Intravenoso Periférico Curto (CIVPC) é o mais utilizado em pacientes internados ou hospitalizados ^(^ 1 ^)^ . 

 Estima-se que 80% dos pacientes hospitalizados utilizam CIVPC ^(^ 2 ^)^ . No entanto, apesar dos benefícios e indicações, há complicações associadas à utilização deste dispositivo. Essas intercorrências podem ser classificadas como locais ou sistêmicas, tais como flebite ^(^ 3 ^)^ , migração de ponta primária e secundária ^(^ 4 ^)^ , Trombose Venosa Profunda (TVP) ^(^ 4 ^)^ , dano no cateter, oclusão ^(^ 3 ^)^ e Infecção da Corrente Sanguínea Associada ao Cateter (ICSAC) ^(^ 1 ^,^ 5 ^)^ . 

 Para pacientes que necessitam de administração de medicamentos incompatíveis por via periférica, de terapia intravenosa por tempo prolongado ou sem condições de punção periférica, pode ser indicado o uso de um Dispositivo de Acesso Vascular Central (DAVC), um deles o *Peripherally Inserted Central Catheter* (PICC) ^(^ 1 ^)^ . 

 O PICC é um cateter longo, flexível, que apresenta indicações específicas de uso, tais como antibioticoterapia acima de seis dias, uso de medicamentos irritantes ou vesicantes, extremos de pH e alta osmolaridade. É inserido por meio de uma veia periférica e alcança posicionamento central no terço distal da junção cavoatrial ^(^ 1 ^)^ . 

 No Brasil, o PICC é, majoritariamente, inserido por enfermeiros devidamente habilitados, e esta prática é legalizada pela Resolução nº 258 do Conselho Federal de Enfermagem (COFEN) ^(^ 6 ^)^ . Contudo, a utilização e aquisição deste dispositivo em hospitais do Sistema Único de Saúde (SUS) ainda é limitada devido ao custo elevado e por aspectos culturais que incluem desconhecimento do cateter e indicações por alguns profissionais ^(^ 7 ^)^ . 

 O uso do PICC é descrito na literatura, principalmente, para população pediátrica e neonatal ^(^ 8 ^-^ 11 ^)^ . Apesar de ser considerado um cateter de primeira escolha, frequentemente enfrenta atrasos na sua inserção, deixando de ser inserido no período correto, o que causa danos importantes e esgotamento da rede venosa periférica nos pacientes hospitalizados ^(^ 12 ^)^ . 

 Diante desse cenário desafiador, surge a importância de compreender as dificuldades enfrentadas na inserção do PICC e as possíveis complicações associadas ao uso deste dispositivo. Trata-se de um conhecimento importante, sobretudo porque o enfermeiro, diante da necessidade de inseri-lo para garantir uma terapia intravenosa segura e eficaz, pode se deparar com a limitação das condições das veias periféricas como fator que impede a adequada inserção ^(^ 1 ^,^ 13 ^)^ . 

Ademais, compreender os fatores que colaboram para o insucesso na inserção ou na incidência de complicações relacionadas ao uso do PICC pode contribuir para que a equipe de saúde atue no sentido de reduzir essas intercorrências. Isso, por sua vez, melhora a qualidade da assistência prestada e favorece a segurança do paciente adulto em uso de PICC, visto que as pesquisas com essa população são escassas. Sendo assim, o objetivo deste estudo foi analisar a ocorrência de dificuldade na inserção periférica do cateter central e a presença de complicações no uso deste dispositivo em pacientes adultos hospitalizados que receberam terapia intravenosa periférica prévia por cateter intravenoso periférico curto, bem como identificar se há associação entre terapia intravenosa periférica e presença de complicações no uso do cateter central inserido perifericamente.

## Método

### Delineamento do estudo

 Estudo de caráter retrospectivo e longitudinal, do tipo coorte. Foram utilizadas as recomendações do *Strengthening the Reporting of Observational Studies in Epidemiology* (STROBE) para reportar a pesquisa. 

### Local

O estudo foi realizado em um hospital de ensino público terciário na cidade de Londrina, PR, Brasil, que possui uma comissão de enfermeiros treinada e especializada na inserção do PICC. Essa comissão segue protocolos institucionais, mas a escolha da veia para a inserção é realizada pelo enfermeiro que realiza o procedimento. Não existiu treinamento para os enfermeiros da comissão durante o período de estudo.

### Período

Os dados foram coletados no período de 01 de janeiro a 31 de dezembro de 2020.

### População

Adultos hospitalizados no período de coleta de dados submetidos a procedimento de inserção de PICC, que receberam terapia intravenosa periférica prévia por cateter intravenoso periférico curto.

### Critérios de seleção

Foram incluídos todos os pacientes com 18 anos ou mais, hospitalizados no período de 01 de janeiro a 31 de dezembro de 2020, que tiveram um PICC inserido com ou sem sucesso e precedido por pelo menos um CIVPC. Foram excluídos aqueles com inserção prévia de um DAVC, bem como os que receberam alta hospitalar com o PICC para uso domiciliar.

### Definição da amostra

A amostra foi definida por conveniência.

### Variáveis do estudo

#### Variável dependente

*Ocorrência de complicação durante o uso do PICC:* ocorrência de pelo menos um evento adverso durante o uso do PICC que não resultou na remoção do dispositivo (migração primária ou secundária, flebite, suspeita de infecção, oclusão total, dano no cateter, ICSAC, trombose venosa, sangramento ou lesão de pele) ^(^ 1 ^)^ . 

#### Variáveis independentes

*Tempo de TIP (variável principal):* duração, em dias, do primeiro dia de uso de CIVPC pelo paciente hospitalizado até a data da inserção do PICC; 

*Migração primária:* situação em que a ponta do PICC não se encontra posicionada na veia cava superior ou na junção cavoatrial ^(^ 1 ^)^ ; 

*Migração secundária:* também chamada de deslocamento, é a saída total da ponta do PICC da veia ^(^ 1 ^)^ ; 

*Flebite:* ocorrência de um ou mais sintomas (dor e/ou sensibilidade no sítio de inserção e no trajeto do PICC, eritema, edema, endurecimento, drenagem purulenta ou cordão venoso palpável) ^(^ 1 ^)^ ; 

*Suspeita de infecção:* sintomas locais ou sistêmicos de infecção atribuídos ao PICC sem confirmação da ICSAC ^(^ 1 ^)^ ; 

*Oclusão total:* um ou mais lúmens do cateter apresentam obstrução intraluminal, não sendo possível injetar líquidos ou aspirar sangue ^(^ 1 ^)^ ; 

*Dano no cateter:* sinais de cateter visivelmente fraturado, vazamento local, disfunção do cateter, evidência radiográfica de extravasamento ou infiltração no tecido ^(^ 1 ^)^ ; 

*ICSAC:* infecção da corrente sanguínea primária em paciente que teve dispositivo de acesso central no período de 48 horas antes do desenvolvimento da infecção da corrente sanguínea sem estar relacionado a uma infecção em outro local ^(^ 1 ^)^ ; 

*Trombose venosa:* neste trabalho foi considerada a TVP, definida pela ocorrência de trombos visualizados a partir de exames de imagem no local do trajeto do PICC em paciente com sintomas de dor no membro cateterizado, edema ou rubor ^(^ 1 ^)^ ; 

*Sangramento na inserção:* presença de sangue visível no local de inserção do dispositivo ^(^ 1 ^)^ ; 

*Lesão de pele:* presença de um ou mais dos sintomas (rubor por mais de 30 minutos após a remoção do curativo, laceração na pele, descamação na pele, bolhas na pele, lesões por trauma, pústula, vesícula, pápula ou ruptura na pele) ^(^ 1 ^)^ ; 

*Tempo de uso do PICC:* considerado o tempo de utilização do PICC, duração em dias a partir da inserção do dispositivo até a remoção; 

*Dificuldade na inserção do PICC:* descrição pelo enfermeiro que realizou a inserção do PICC em prontuário ou em ficha específica sobre a presença de alguma dificuldade no procedimento de inserção; 

*Número de punções:* quantitativo de punções no procedimento de inserção do PICC; 

*Remoção do PICC:* último dia de uso do PICC, data da remoção; 

*Unidade de internação:* local de internação hospitalar no momento da inserção do PICC (pronto socorro, Unidade de Terapia Intensiva (UTI) adulto, unidade de clínica médica cirúrgica, centro cirúrgico, centro de tratamento de queimados e UTI de queimados); 

*Punções venosas periféricas prévias ao PICC* : quantidade de CIVPC durante a TIP; 

*Medicamentos vesicantes e irritantes utilizados na TIP* : considerado medicamento vesicante aquele capaz de causar bolhas, descamação ou necrose quando há extravasamento ^(^ 1 ^)^ ; 

*Local de inserção do PICC:* sítio de inserção do PICC, as veias basílica, cefálica, axilar ou outras foram classificadas como membros superiores, e jugular externa; 

*Remoção do PICC causada por complicação* : migração primária ou secundária, flebite, suspeita de infecção, dano no cateter, oclusão total, ICSAC, TVP, sangramento ou lesão de pele que culminaram na remoção do PICC. 

### Instrumentos utilizados para a coleta das informações

Utilizou-se um formulário elaborado pelo núcleo de pesquisa do autor, com informações acerca da TIP, condições da rede venosa, múltiplas punções periféricas, uso de medicamentos vesicantes e caracterização do paciente para coleta dos dados do prontuário. Além deste, foi utilizado um formulário dos procedimentos de inserção do PICC, com informações sobre indicação da inserção do dispositivo, dados do PICC (material, calibre, lumens e comprimento), local de inserção, vaso acessado, condições da rede venosa que dificultam a punção do vaso e introdução do cateter, dificuldade na inserção, radiografia controle e registro de utilização de ultrassom e dados sobre a retirada (motivo da retirada, integridade do cateter, culturas de ponta de cateter e hemocultura).

### Coleta de dados

Na coleta de dados foram consultados os prontuários dos pacientes e os formulários dos procedimentos de inserção do PICC daqueles hospitalizados no período de 01 de janeiro a 31 de dezembro de 2020. Os formulários dos procedimentos de inserção do PICC foram preenchidos pelos enfermeiros da comissão de terapia infusional do hospital.

### Tratamento e análise dos dados

Inicialmente os dados foram descritos através de frequências absolutas e percentuais (variáveis qualitativas) e por meio de medidas como média, desvio-padrão, mínimo, mediana e máximo (variáveis quantitativas). Para estimar o Risco Relativo para os desfechos de dificuldade na inserção, complicações na internação, deslocamento e remoção do PICC em relação às variáveis de interesse, utilizou-se o modelo de regressão de Poisson com variância robusta simples e múltiplo (quando adequado). O teste de Log-rank foi aplicado para verificar evidências de diferenças entre as curvas de sobrevida. As análises foram realizadas com auxílio do software SAS 9.4. Neste trabalho, adotou-se nível de significância de 5% (p < 0,05) e foram aplicados os testes de Kolmogorov-Smirnov ou Shapiro-Wilk para a normalidade e homoscedasticidade.

## Aspectos éticos

 O estudo foi submetido e aprovado pelo Comitê de Ética em Pesquisa (CEP) da Escola de Enfermagem de Ribeirão Preto (EERP) e pelo CEP do Hospital Universitário de Londrina (HUL), sob parecer nº 3.627.165 e CAAE 16451019.8.0000.5393, conforme Resolução nº 466/2012 ^(^ 14 ^)^ . Solicitou-se e obteve-se a dispensa do Termo de Consentimento Livre e Esclarecido. 

## Resultados

### Participantes

Dos 105 pacientes adultos hospitalizados submetidos à inserção do PICC, 28 foram excluídos por não possuírem ao menos um CIVPC antes do PICC e outro por alta para tratamento domiciliar com o dispositivo. Compuseram a amostra final 76 pacientes, dos quais 59 (77,6%) tiveram a inserção do PICC realizada com sucesso e 17 (22,3%) sem sucesso.

A idade média na internação foi de 50 anos (desvio-padrão 18,29), no mínimo 18 e no máximo 90 anos (mediana 47 anos), enquanto o sexo de maior prevalência foi o masculino (69,74%).

Sobre as unidades de internação, mais da metade (51,3%) encontrava-se em unidade de clínica médico-cirúrgica. Uma particularidade deste estudo é a internação na UTI de Queimados, que chegou a 15,8%. Em seguida, 21,0% dos pacientes estavam internados na UTI adulto.

#### Terapia intravenosa periférica antes do PICC

A média de tempo em dias que os pacientes fizeram uso de CIVPC foi de 10,53 (desvio-padrão 31,58), com mediana de 4 dias, e máximo de tempo de 274 dias. A média de números de punções venosas prévias ao PICC foi de 4,22 (desvio-padrão 3,74), mediana de três, no mínimo uma e máximo de 8.

Também foi analisado o uso de medicamentos irritantes e vesicantes neste período, revelando que 72,3% dos participantes utilizaram este tipo de medicamento. Os mais recorrentes foram: Cloreto de Potássio (48,2%), Cloridrato de Vancomicina (29,0%) e Piperacilina sódica + Tazobactam sódico (25,4%).

Não houve associação do tempo de TIP (a cada aumento de um dia) comparado com a dificuldade na inserção do PICC nos modelos bruto (RR 0,99; IC 95% 0,98-1,00; Valor p = 0,23) e ajustado por sexo e idade (RR 1,00; IC 95% 0,99-1,00; Valor-p = 0,21).

Para a administração de medicamentos irritantes e vesicantes durante a TIP, também não foi observada associação no modelo de regressão de Poisson bruto (RR 0,89; IC 95% 0,52-1,54; Valor-p = 0,69) e ajustado por sexo e idade (RR 1,21; IC 95% 0,68-2,16; Valor-p = 0,53) comparado à dificuldade na inserção do PICC.

#### Indicação do uso e inserção do PICC

O tempo médio até a inserção do PICC neste estudo foi de 10,88 dias (desvio-padrão 13,06), no mínimo zero e máximo de 73 dias e mediana de 6 dias, considerando o primeiro dia de internação até a data da inserção do PICC. A indicação de uso mais descrita foi para a administração de antimicrobianos (78,9%). O local de inserção mais utilizado pelos enfermeiros foi a jugular externa (54,6%). Sobre o tempo de uso do PICC, detectou-se média de 9,24 dias (desvio-padrão 12,58), no mínimo zero e máximo de 78 dias (mediana de 6 dias).

Mais da metade dos procedimentos ocorreu sem dificuldade na inserção do PICC (56,0%). A média de número de punções para a inserção do PICC foi de 1,78 (desvio-padrão 1,02), no mínimo uma e máximo de seis. A ultrassonografia foi realizada para auxílio na inserção de 2 (2,6%) PICC. A taxa de sucesso do procedimento foi de 97,6% entre os que não tiveram dificuldade na inserção do PICC e de 51,5% entre os que apresentaram essa dificuldade.

Sobre as características do PICC, 71 (97,2%) dos cateteres foram do tipo monolúmen e 68 (93,1%) de composição de poliuretano, fatores influenciados pela licitação de compra daquele período.

 Na Regressão de Poisson ( Tabela 1 ), houve evidências de associação entre o número de punções e a dificuldade na inserção do PICC. Pelo modelo ajustado por sexo e idade, estima-se que, em média, a cada aumento de uma punção, o risco de dificuldade aumente em 42% (RR 1,42; IC 95% 1,11-1,81). Não houve associação do local da inserção (jugular externa ou membros superiores) com a dificuldade na inserção do PICC (p=0,18). 

**Tabela 1 -:** Modelos bruto e ajustado de regressão logística da dificuldade de inserção do *Peripherally Inserted Central Catheter* (PICC), com cálculo do risco relativo (n = 76). Londrina, PR, Brasil, 2020

**Comparação com a dificuldade de inserção do PICC** *	**Bruto**				**Ajustado**			
	**Risco relativo**	**Intervalo de confiança (95%)**		**Valor p**	**Risco relativo** ^†^	**Intervalo de confiança (95%)**		**Valor p**
Jugular Externa vs. Membros Superiores	0,48	0,28	0,84	<0,01	0,67	0,38	1,20	0,18
Número de Punções (a cada aumento de uma punção)	1,57	1,29	1,92	<0,01	1,42	1,11	1,81	<0,01

* *Peripherally Inserted Central Catheter*

^†^ Modelo ajustado por sexo e idade (*n*= 33)

#### Complicações no uso do PICC

 As complicações apresentam relevância neste estudo e foram presentes em 72,8% dos pacientes em uso do PICC. Não foi verificada associação de presença de complicação no uso do PICC com o tempo de TIP, tanto no modelo bruto (RR 0,99; IC 95% 0,97-1,00; Valor-p 0,08) quanto no ajustado (RR 0,99; IC 95% 0,98-1,00; Valor-p 0,08) ( Tabela 2 ). 

 Na Tabela 3 é possível observar que a migração de ponta secundária foi a mais referida (53,4%), seguida de flebite (16,2%) e migração de ponta primária (11,6%). Importante citar que, para as Tabelas 3, 4 e 5, foram consideradas as inserções com sucesso (n=59), visto que a análise descritiva teve o objetivo de descrever as complicações do uso do PICC. 

 Mais da metade dos pacientes apresentou complicações que levaram à remoção do PICC (54,2%), majoritariamente por migração de ponta secundária (62,5%), como demonstra a Tabela 4 . O tempo médio, em dias, entre a presença de complicação e remoção foi de 7,6 (desvio-padrão 12,8), no mínimo zero e máximo de 73 dias. O tempo médio livre de complicação foi de 8,8 dias. 

Para os participantes do estudo que apresentaram algum tipo de complicação no uso do PICC, a maior parte (78,3%) teve a inserção do cateter em veia jugular externa comparada aos membros superiores. A média de idade na internação foi maior (53,51 anos) entre os pacientes que apresentaram complicação no uso do PICC.

 A Tabela 2 mostra a associação no modelo bruto de Poisson para o tempo de uso do PICC a cada aumento de um dia em relação à incidência de complicação (RR 1,01; IC 95% 1,0009-1,01; Valor-p 0,02). 

**Tabela 2 -:** Modelos bruto e ajustado de regressão logística da presença de complicações no uso do *Peripherally Inserted Central Catheter* (PICC), com cálculo do risco relativo (n = 59). Londrina, PR, Brasil, 2020

**Comparação com a presença de complicações no uso do PICC** *	**Bruto**				**Ajustado**			
	**Risco relativo** ^†^	**Intervalo de confiança (95%)**		**Valor p**	**Risco relativo** ^†^	**Intervalo de confiança (95%)**		**Valor p**
Tempo de Terapia Intravenosa Prévia: a cada aumento de um dia	0,99	0,97	1,00	0,08	0,99	0,98	1,00	0,08
Tempo de uso do PICC*: a cada aumento de um dia	1,01	1,0009	1,01	0,02	1,01	0,998	1,02	0,12

^*^ *Peripherally Inserted Central Catheter*

^†^ Modelo ajustado controlado por sexo, idade e unidade de internação ( *n* = 43)

**Tabela 3 -:** Descrição das variáveis relacionadas às complicações no uso do *Peripherally Inserted Central Catheter* (PICC) em pacientes adultos hospitalizados (n = 59). Londrina, PR, Brasil, 2020

**Variáveis**	**n**	**%**
**Presença de complicação**		
Sim	43	72,8
Não	16	27,1
**Migração secundária**		
Sim	31	70,4
**Flebite**		
Sim	13	30,2
**Suspeita de infecção**		
Sim	8	18,6
**Migração primária**		
Sim	6	13,9
**Oclusão total**		
Sim	5	11,6
**Sangramento**		
Sim	5	11,6
**Dano no cateter**		
Sim	4	9,3
**Infecção de corrente sanguínea associada ao cateter**		
Sim	1	2,3
**Lesão de pele**		
Sim	1	2,3
**Trombose venosa**		
Sim	0	0,0

**Tabela 4 -:** Descrição das variáveis relacionadas à remoção por complicação do *Peripherally Inserted Central Catheter* (PICC) em pacientes adultos hospitalizados (n = 59). Londrina, PR, Brasil, 2020

**Variáveis**	**n**	**%**
**Remoção do PICC** * **durante a internação causada por complicação**		
Sim	32	54,2
Não	27	45,8
**Migração secundária**		
Sim	20	62,6
**Suspeita de infecção**		
Sim	6	18,8
**Dano no cateter**		
Sim	2	6,2
**Oclusão total**		
Sim	2	6,2
**Flebite**		
Sim	1	3,1
**Infecção de corrente sanguínea associada ao cateter**		
Sim	1	3,1

* *Peripherally Inserted Central Catheter*

 Na análise de Poisson com variância robusta apresentada na Tabela 5 , houve evidência de associação entre o local da inserção do PICC e a sua remoção por complicações, sendo que a inserção na jugular externa apresentou maior risco de remoção por complicação em relação aos membros superiores (RR 2,12; IC 95% 1,11-4,07; Valor-p 0,02). Considerando o desfecho migração secundária, não houve evidência de associação com o local de inserção. 

**Tabela 5 -:** Modelo de regressão de Poisson com variância robusta e cálculo do risco relativo (n = 59). Londrina, PR, Brasil, 2020

**Variável**	**Local**					
	**Jugular Externa**	**Membros Superiores**	**Risco Relativo** **(Jugular Externa** *vs* **Membros)**	**Intervalo de confiança (95%)**		**Valor p**
Remoção do PICC* por complicações (n=59)						
Não	12 (32,4)	15 (68,2)				
Sim	25 (67,6)	7 (31,8)	2,12	1,11	4,07	0,02
Complicação: migração secundária (n=43)						
Não	6 (23,3)	6 (42,9)				
Sim	23 (76,7)	8 (57,1)	1,34	0,82	2,20	0,24

^*^ *Peripherally Inserted Central Catheter*

## Discussão

 A abordagem deste estudo em associar a TIP ao uso e a complicações do PICC é original. As sucessivas falhas no estabelecimento de um acesso venoso por CIVPC levam a diversas tentativas de punção que apresentam múltiplos impactos, inclusive financeiros ^(^ 7 ^)^ . 

 A preservação das veias periféricas durante a hospitalização é crucial para reduzir complicações, garantir a segurança e promover a satisfação dos pacientes ^(^ 15 ^)^ . No entanto, observou-se que o número de punções periféricas antes da inserção do PICC impactou negativamente nas taxas de sucesso na primeira tentativa das inserções do PICC ^(^ 12 ^,^ 16 ^-^ 18 ^)^ . Acredita-se que tal achado pode estar relacionado à indicação tardia do PICC, que contribui para a escolha de veias não recomendadas, como a jugular externa. 

 A infusão de medicamentos vesicantes e irritantes por meio do CIVP também coopera para a fragilidade vascular. Apesar da infusão periférica recorrente, administrá-los dessa forma é contraindicado e associado a complicações do CIVP ^(^ 18 ^-^ 19 ^)^ . Neste estudo, não foi identificada associação da administração desses fármacos com a dificuldade de inserção do PICC. 

 A alta frequência de inserção do PICC em veia jugular externa foi uma particularidade deste estudo, de forma incongruente com as recomendações de *guidelines* nacional e internacionais ^(^ 1 ^,^ 20 ^)^ . Na literatura não foi identificada a inserção do PICC em veia jugular externa. A primeira veia de escolha para punção e inserção do PICC deve ser a basílica, e as veias acima da fossa antecubital são as mais apropriadas para a inserção do PICC, achado encontrado em diversos estudos, inclusive nacionais ^(^ 21 ^-^ 23 ^)^ . Reitera-se que a inserção em veia jugular externa aumentou o risco de remoção e dificuldade na inserção do PICC em mais de uma vez, comparada aos membros superiores. 

 Tradicionalmente, a punção em veia jugular externa apresenta indicações específicas, incluindo pacientes que não apresentam outras veias mais evidentes, por exemplo em membros superiores, ou quando não há disponibilidade de tecnologia de visualização vascular ^(^ 24 ^)^ . Anatomicamente, trata-se de uma veia mais saliente e visível que as demais ^(^ 24 ^)^ , como o uso do ultrassom foi utilizado em apenas duas punções e a rede venosa periférica estava prejudicada, foi possivelmente o motivo pelo qual, a veia jugular se tornou única opção viável e de preferência para a inserção de cateteres, neste caso, o PICC. 

 Não foi evidenciada associação do local da inserção (jugular externa ou membros superiores) com a dificuldade na inserção do PICC, conforme Tabela 1 . Entretanto, pondera-se que, por fatores anatômicos e pelo esgotamento da rede venosa periférica, a veia jugular externa esteja mais preservada. Além disso, o enfermeiro, com vistas ao êxito do procedimento, possivelmente tenha mais sucesso no ato da inserção nesta veia em comparação com as demais dos membros superiores, as quais se encontram em estado frágil e apresentam maiores complicações na inserção pela visualização reduzida ^(^ 13 ^)^ . 

 Embora utilizados nesta área anatômica, a fixação do PICC e de outros DAVC em veias jugulares externas tornou-se um desafio que inspira diversos cuidados de enfermagem. Complicações como migração e fixação do curativo podem culminar na remoção do PICC, uma vez que o pescoço é uma área de grande movimentação, diaforese e crescimento de pelos ^(^ 25 ^)^ . 

 A Tabela 5 evidenciou que a migração secundária não apresentou associação para a inserção do PICC em jugular externa na comparação com os membros superiores. Contudo, ainda na Tabela 5 , a remoção do PICC causada por complicações esteve associada ao local de inserção em jugular externa, mostrando-se verídica a dificuldade de cuidado do PICC nesta região. 

 Diversos fatores podem contribuir para dificuldades ou sucesso do procedimento de inserção do PICC. Um dos mais importantes é a fragilidade vascular, que acentua a importância da preservação das veias periféricas ^(^ 13 ^)^ . Neste estudo, a jugular externa foi o local de inserção mais utilizado. Acredita-se que, por favorecer a visualização, tal veia facilite a punção. 

 Como alternativa a esta difícil visualização e palpação dos vasos, a utilização da ultrassonografia torna-se extremamente eficaz para o sucesso do procedimento e redução das complicações ^(^ 1 ^)^ . No entanto, no presente estudo, duas inserções foram guiadas por ultrassom. 

 Tais tecnologias de visualização vascular, além de propiciarem maior sucesso no procedimento por evitarem as diversas tentativas de punção sem sucesso, causam conforto na comparação com técnicas convencionais de punção direta ^(^ 26 ^)^ . Identificou-se que, ao aumentar o número de punções, intensificou-se o risco de dificuldade na inserção. 

 A indicação do uso do PICC permanece majoritariamente para antibioticoterapia, corroborando a literatura existente ^(^ 22 ^,^ 27 ^)^ . O tempo médio de uso do PICC neste estudo mostrou-se apropriado de acordo com recomendações ^(^ 1 ^,^ 28 ^)^ . Pesquisas apontam menores prevalências de complicações no uso do PICC ^(^ 21 ^-^ 22 ^,^ 29 ^)^ , com achado importante neste estudo do deslocamento de ponta secundária. 

 Tendo em vista que as complicações relacionadas ao PICC podem resultar na necessidade de remoção do dispositivo, um dos desfechos causados é a interrupção e consequente atraso da terapêutica. A migração primária ou secundária deste estudo foi muito maior em relação aos achados de pesquisas prévias ^(^ 4 ^,^ 21 ^-^ 23 ^)^ . No entanto, o hospital em questão, assim como diversos do cenário brasileiro, não possuía a padronização de dispositivos de estabilização que diminuem o risco de deslocamento devido ao alto custo ^(^ 1 ^)^ . 

A amostra por conveniência foi um fator limitante deste estudo. De forma semelhante aos demais hospitais públicos do país, os processos de licitação interferem na disponibilidade dos PICC e na modificação das marcas e composição dos materiais.

Este estudo apoia a compreensão do enfermeiro sobre a inserção do PICC, destacando a importância do conhecimento deste profissional sobre a preservação da rede venosa periférica previamente à sua inserção e indicação adequada do cateter, além de identificar complicações no uso deste dispositivo. Ao enfatizar a importância do discernimento clínico do enfermeiro, este estudo visa contribuir para a otimização do processo de inserção do PICC e redução de complicações associadas.

## Conclusão

Discussões sobre a preservação da rede venosa periférica e indicação do cateter adequado durante a TIP do paciente adulto hospitalizado ainda são pouco exploradas na literatura. Neste estudo, infelizmente, identificou-se alta prevalência de administração de medicamentos vesicantes durante a TIP, o que pode culminar no esgotamento da rede vascular, porém sem associação estatística. Quanto ao tempo de TIP, não houve associação com a presença de complicações no uso do PICC.

A migração secundária foi relatada como a principal complicação durante o uso do PICC e causa mais prevalente de remoção por complicação.

Acredita-se que a presença de veias fragilizadas e comprometidas dificulte o procedimento de inserção do PICC, podendo estar relacionada ao atraso da indicação do PICC.

Verificou-se também que o número de punções para a inserção do PICC aumenta a dificuldade de inseri-lo.

De modo contraditório aos achados nacionais e internacionais, a inserção do PICC em veia jugular externa foi recorrente. Ademais, foi evidenciado que a inserção na jugular externa apresenta maior risco de remoção por complicação em relação aos membros superiores. Não houve associação do local da inserção (jugular externa ou membros superiores) com a dificuldade na inserção do PICC.

A escassez de estudos que relacionem a indicação e utilização do PICC em pacientes adultos ressalta a importância desta pesquisa e a necessidade do desenvolvimento de práticas de saúde seguras, bem como de publicações na temática em questão.
